# Supplementary material for: Loss of β-cell identity and diabetic phenotype in mice caused by disruption of CNOT3-dependent mRNA deadenylation
Source: Commun Biol. 2020 Aug 28;3:476. doi: 10.1038/s42003-020-01201-y (PMC7455721; doi:10.1038/s42003-020-01201-y)
Supplement: Supplementary file 1 — Supplementary Information [file 42003_2020_1201_MOESM1_ESM.pdf]

Supplementary information for:

Loss of β-cell identity and diabetic phenotype in mice caused by disruption of CNOT3-dependent mRNA deadenylation

Mostafa et al.

Supplementray Figures:

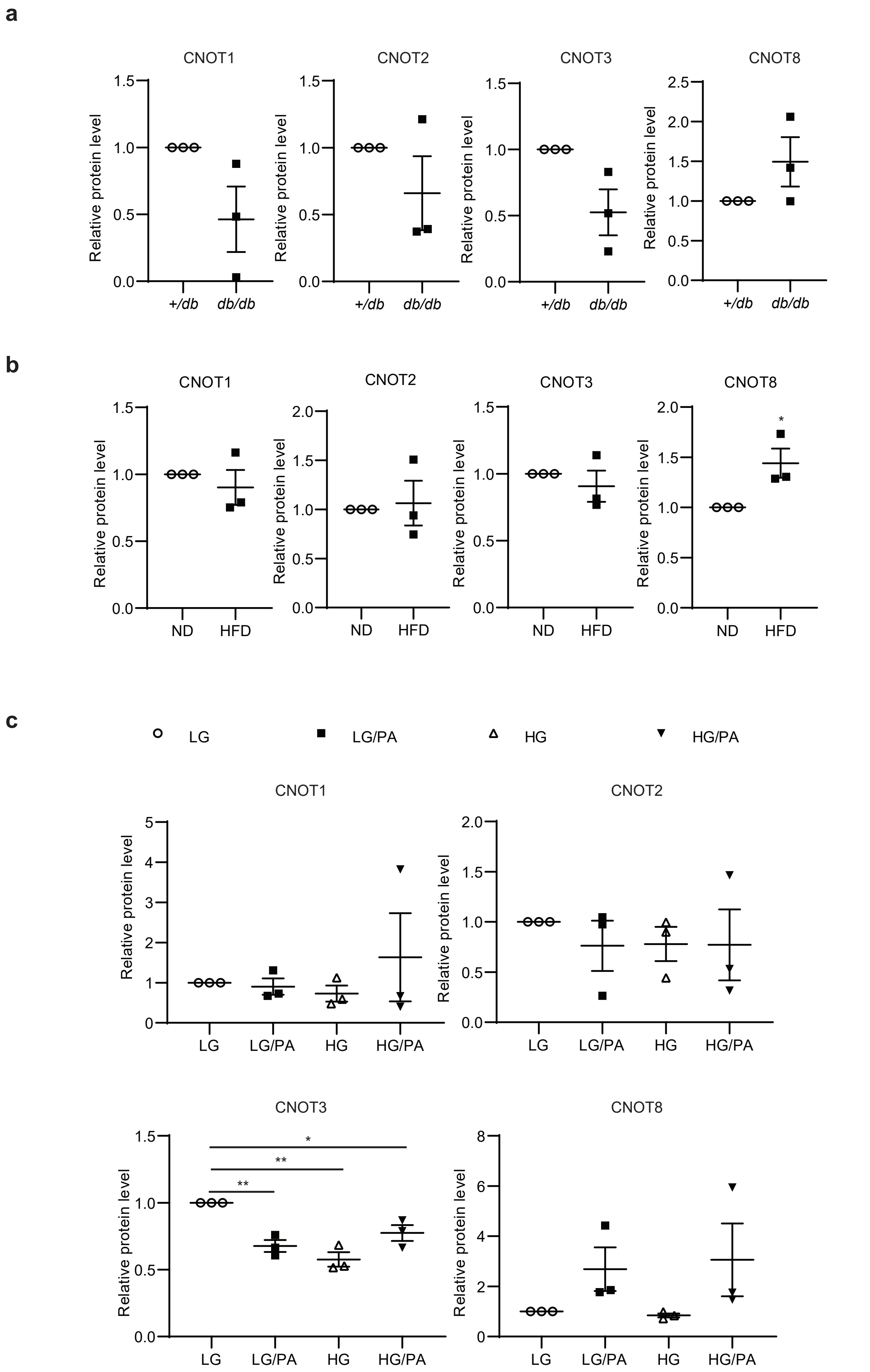

Supplementary Fig.1 : Band quantification of immunoblots of CCR4-NOT complex subunits in Fig.1 (n=3).
Band intensities in *+/db*, ND and LG lanes were set to 1. Data are presented as mean ± S.E.M; * $P < 0.05$; ** $P < 0.01$; *** $P < 0.001$, two-tailed Student's *t*-test.

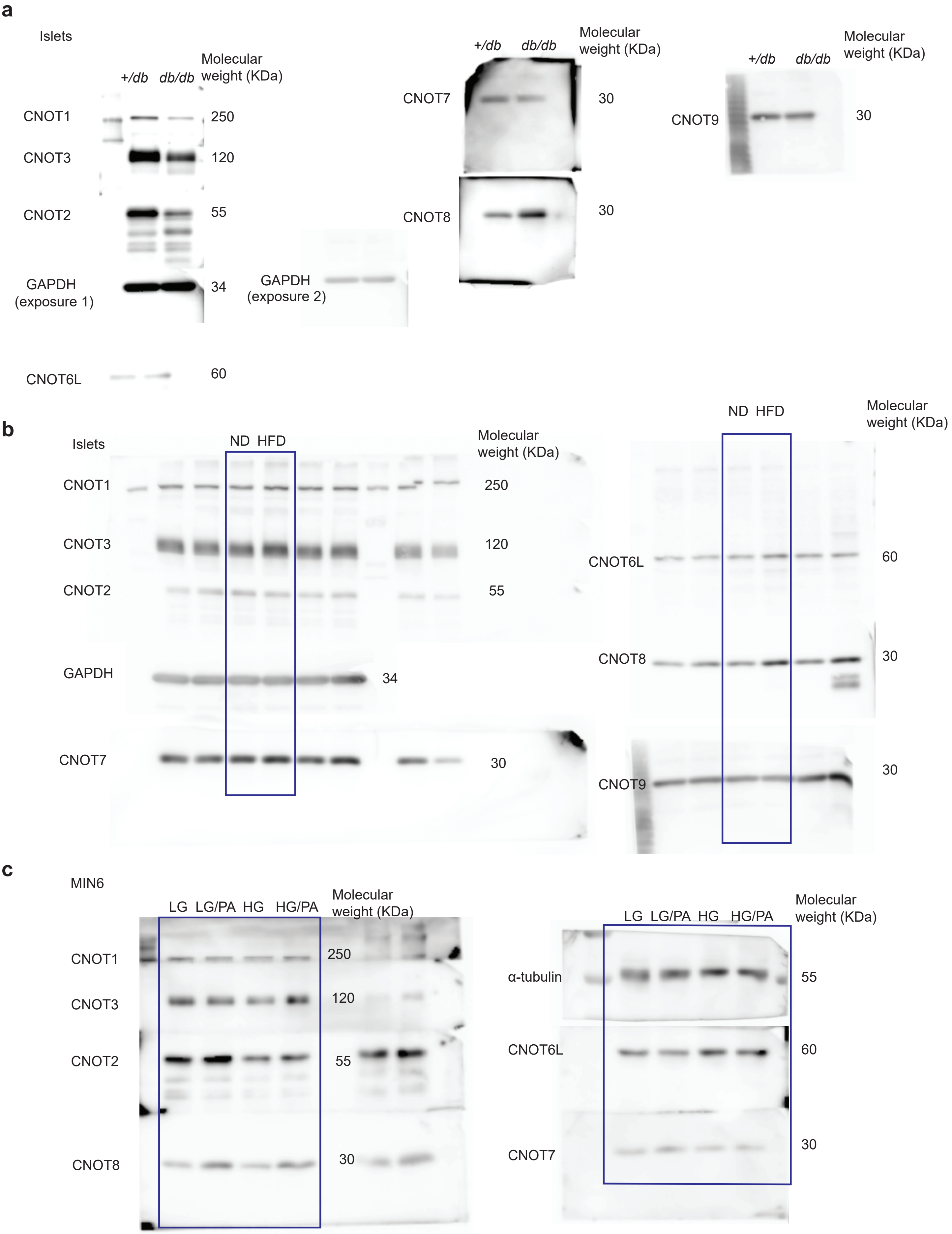

Supplementary Fig. 2:Annotated membrane images of immunoblot analysis of CCR4-NOT complex subunits in : (a) islet lysates from 16-week-old +/db control and db/db mice. (b) islet lysates from 20-week-old mice fed a normal diet (ND) or a high fat diet (HFD) for 12 weeks. (c) MIN6 cells under low/high glucose conditions (LG/HG) with or without palmitic acid (PA) treatment.
Each panel is one experiment representative of three independent experiments (n=3).

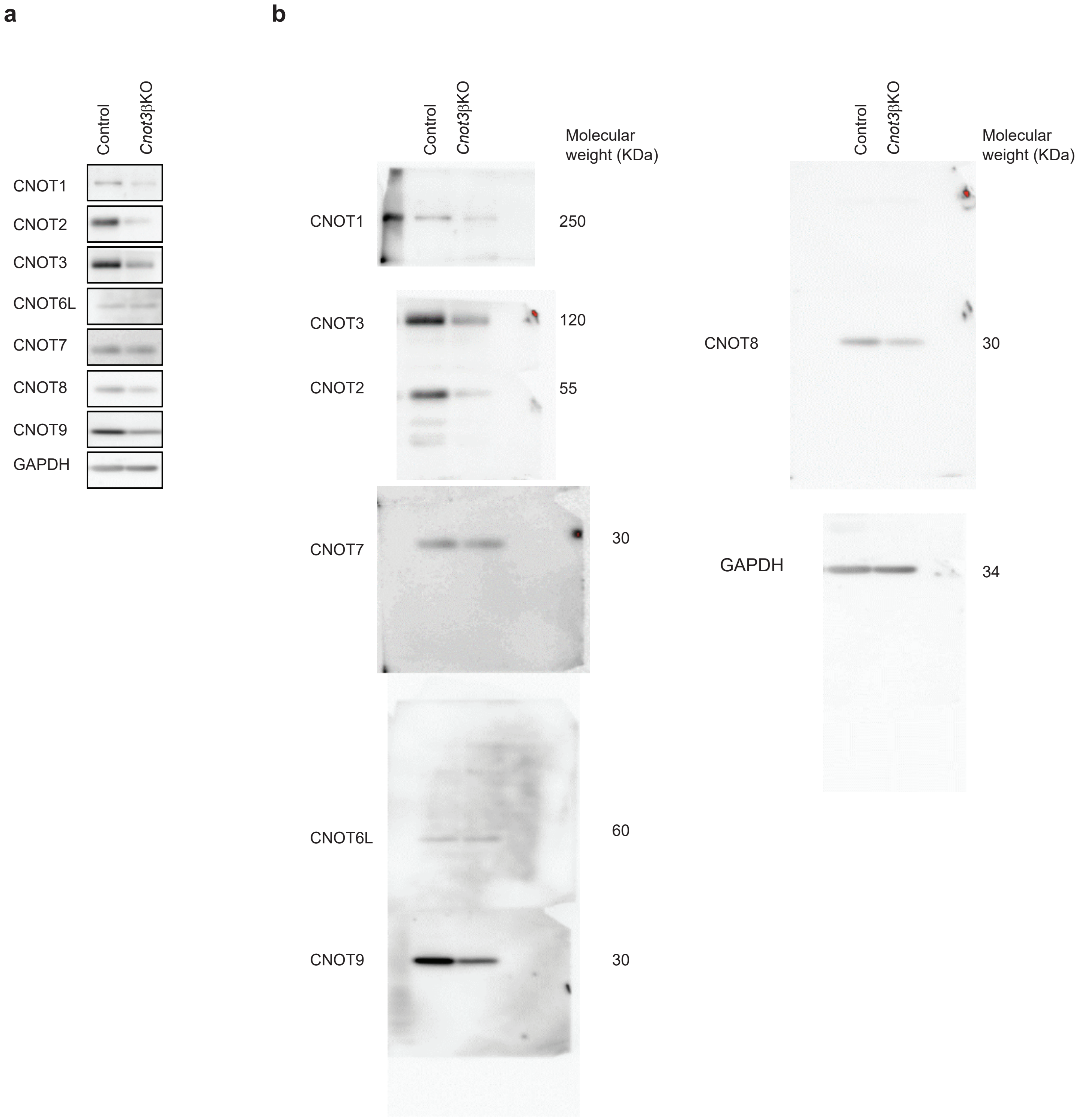

Supplementary Fig. 3:
(a) Immunoblot analysis of CCR4-NOT subunits in islets after β cell specific suppression of CNOT3.
(b) Annotated membrane images of immunoblot analysis of CCR4-NOT complex subunits in islet lysates from control and *Cnot3*βKO mice. This figure is one experiment representative of three independent experiments (n=3).

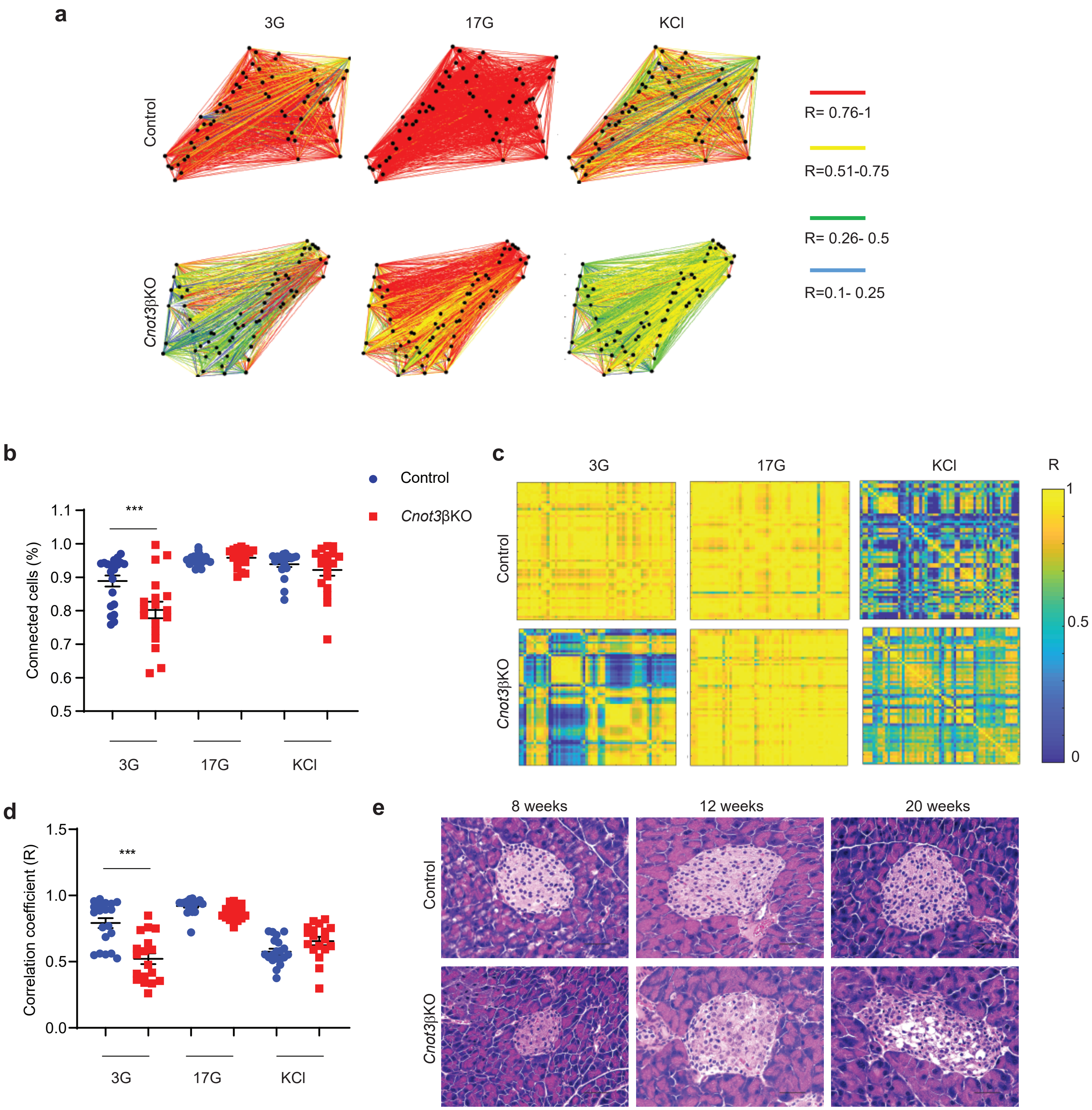

Supplementary Fig. 4:
(a) Cartesian functional connectivity maps displaying the x-y position of analysed cells (black dots). Cells are connected with a line where the strength of each cell pair correlation (the Pearson R statistic) is colour coded: red for R of 0.76 to 1.0, yellow for R of 0.51 to 0.75, green for R of 0.26-0.5 and blue for R of 0.1 to 0.25. Results are shown at low (3G), high (17G) glucose or 20mM KCl for each of the mice groups during the imaging period.
(b) The proportion of significantly connected cell pairs was reduced significantly (p<0.001) at basal glucose levels (3G) in islets from *Cnot3*βKO (n=18) mice compared to those from control (n=20 islets) mice.
(c) Heat maps showing the Pearson coefficient of each cell pair in a colour-coded manner (no correlation; dark blue (0), high correlation; yellow (1)). Results are shown at low (3G), high (17G) Glucose or 20mM KCl for each of the mice groups during the imaging period.
(d) Examined at low glucose, the correlation coefficient (R) value between β cell pairs reduced significantly (P<0.0001) following *Cnot3* deletion.
(e) H & E staining of control and *Cnot3*βKO pancreas sections from 8-, 12- and 20- week old mice.
Data are presented as mean ± S.E.M; * *P* < 0.05; ** *P* <0.01; *** *P* <0.001, one-way ANOVA with post-hoc Tukey test.

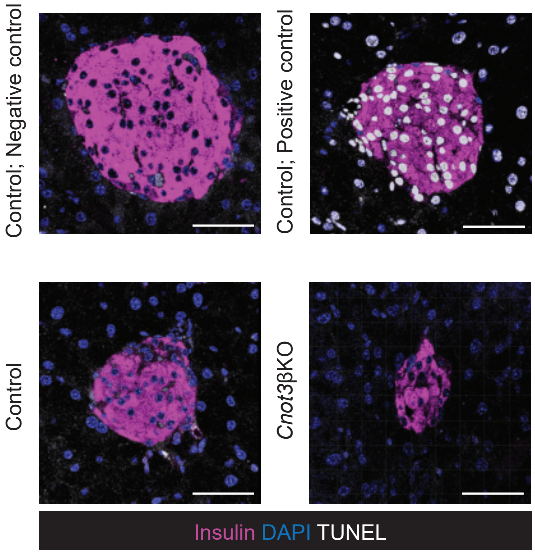

Supplementary Fig. 5: TUNEL assay performed on pancreatic sections from 8-week-old control and *Cnot3*βKO male mice revealing no obvious β cell death in *Cnot3*βKO mice. Negative control: Pancreatic section from control mouse treated with labeling solution without terminal transferase. Positive control: Pancreatic section from control mouse treated with DNase. A white scale bar represents 50 μm.

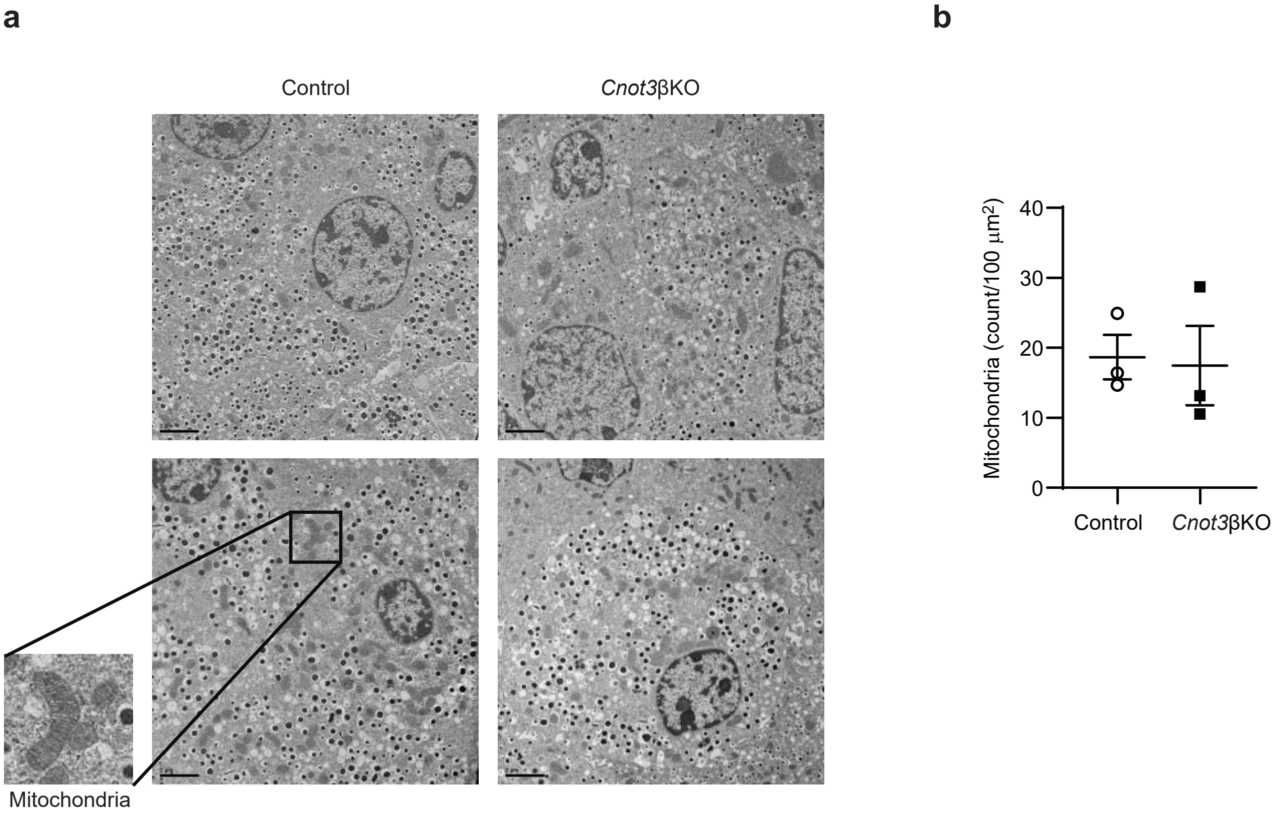

Supplementary Fig. 6: (a) Transmission electron microscopy performed on islets isolated from 8-week-old control and *Cnot3*βKO mice. We show an enlarged example of the mitochchondria. A black scale bar represents 2 μm.
(b) Number of mitochondria per 100 μm^2. Each point represents the mean of mitochondrial count per 100 μm^2 in each biological replicate (n=3).

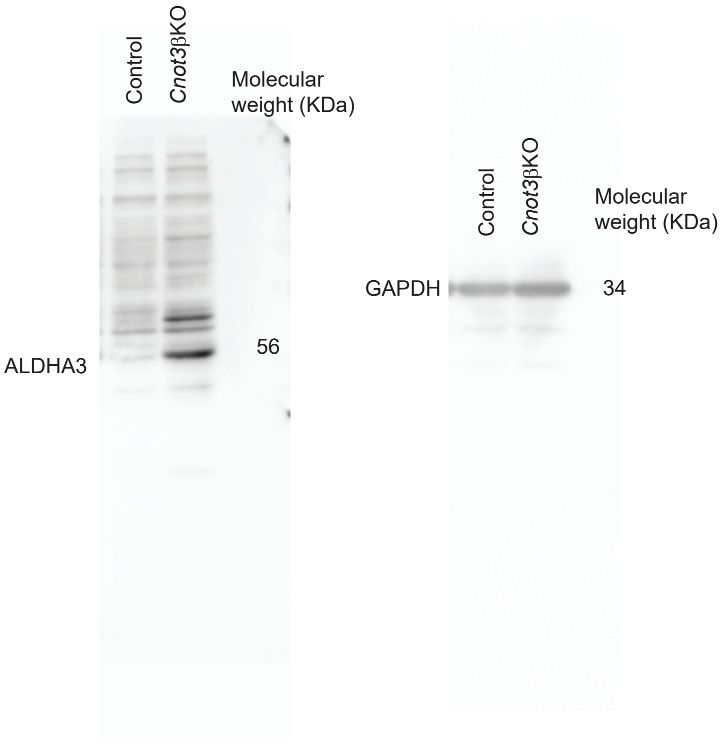

Supplementary Fig. 7: Annotated membrane images of immunoblot analysis of ALDH1A3 in islet lysates from control and *Cnot3*βKO mice.

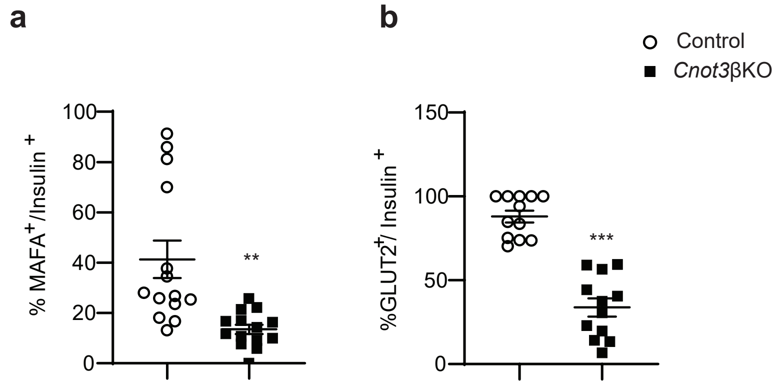

Supplementary Fig. 8: Quantification data of immunofluorescence analysis presented in Fig. 4e. Each data point represents (a) %MAFA⁺ in Insulin⁺ β cells in control and *Cnot3*βKO islets (n=14) and (b) %GLUT2⁺ in Insulin⁺ β cells in control and *Cnot3*βKO islets (n=12) from 3 mice per genotype.
Data are presented as mean ± S.E.M; * $P < 0.05$; ** $P < 0.01$; *** $P < 0.001$, two-tailed Student's *t*-test.

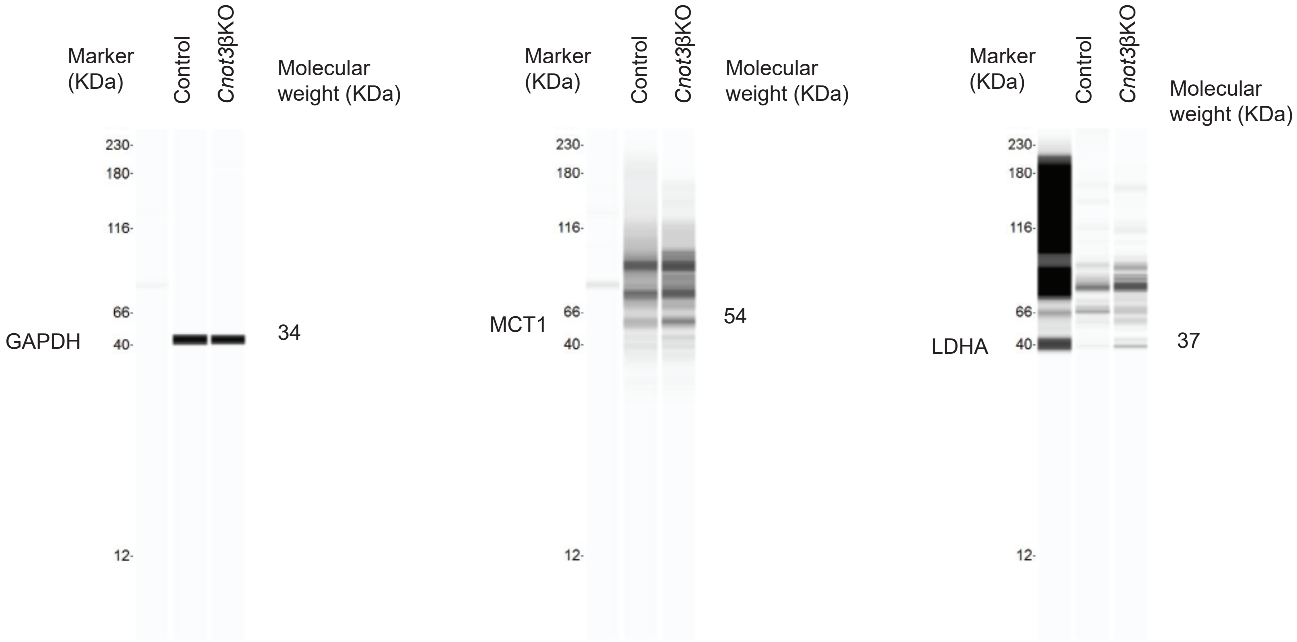

Supplementary Fig. 9: Annotated lane images of Simple Wes analysis of MCT1 and LDHA in islet lysates from control and *Cnot3*βKO mice. Each panel is one experiment representative of three independent experiments (n=3).

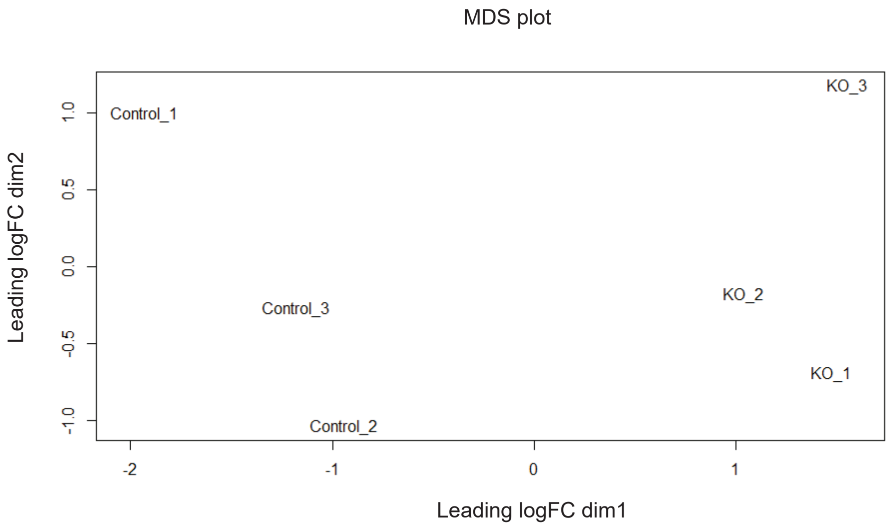

Supplementary Fig. 10: Multidimensional scaling (MDS) plot of 3 control and 3 *Cnot3*βKO (KO) samples.

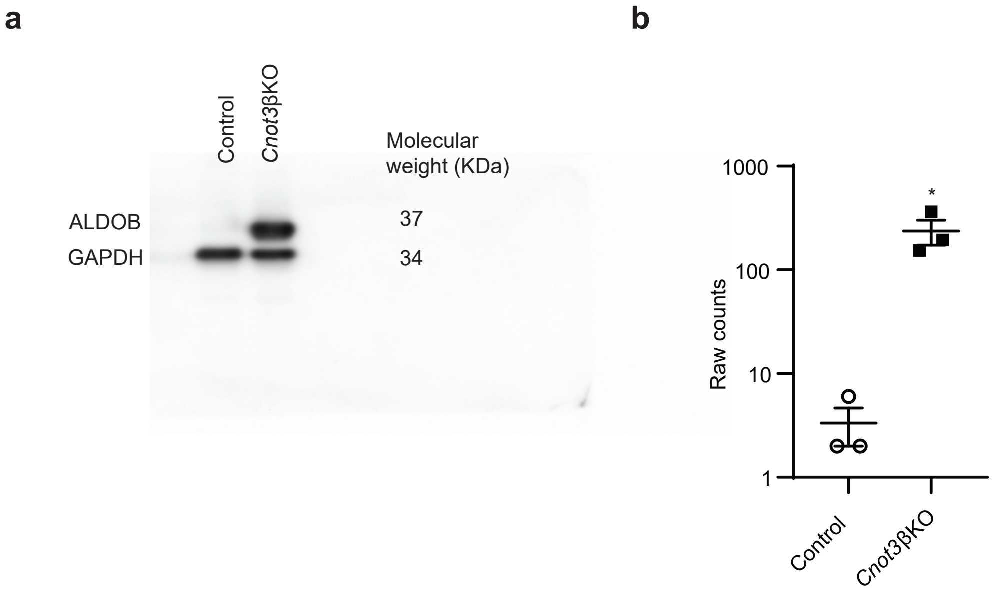

Supplementary Fig. 11: (a) Annotated membrane image of immunoblot analysis of ALDOB in Control and *Cnot3*βKO islets.
This is one experiment representative of three independent experiments (n=3).
(b) Intron raw count of Aldob (n=3), Data are presented as mean ± S.E.M; * *P* < 0.05; ** *P* <0.01; *** *P* <0.001, two-tailed Student's *t*-test.

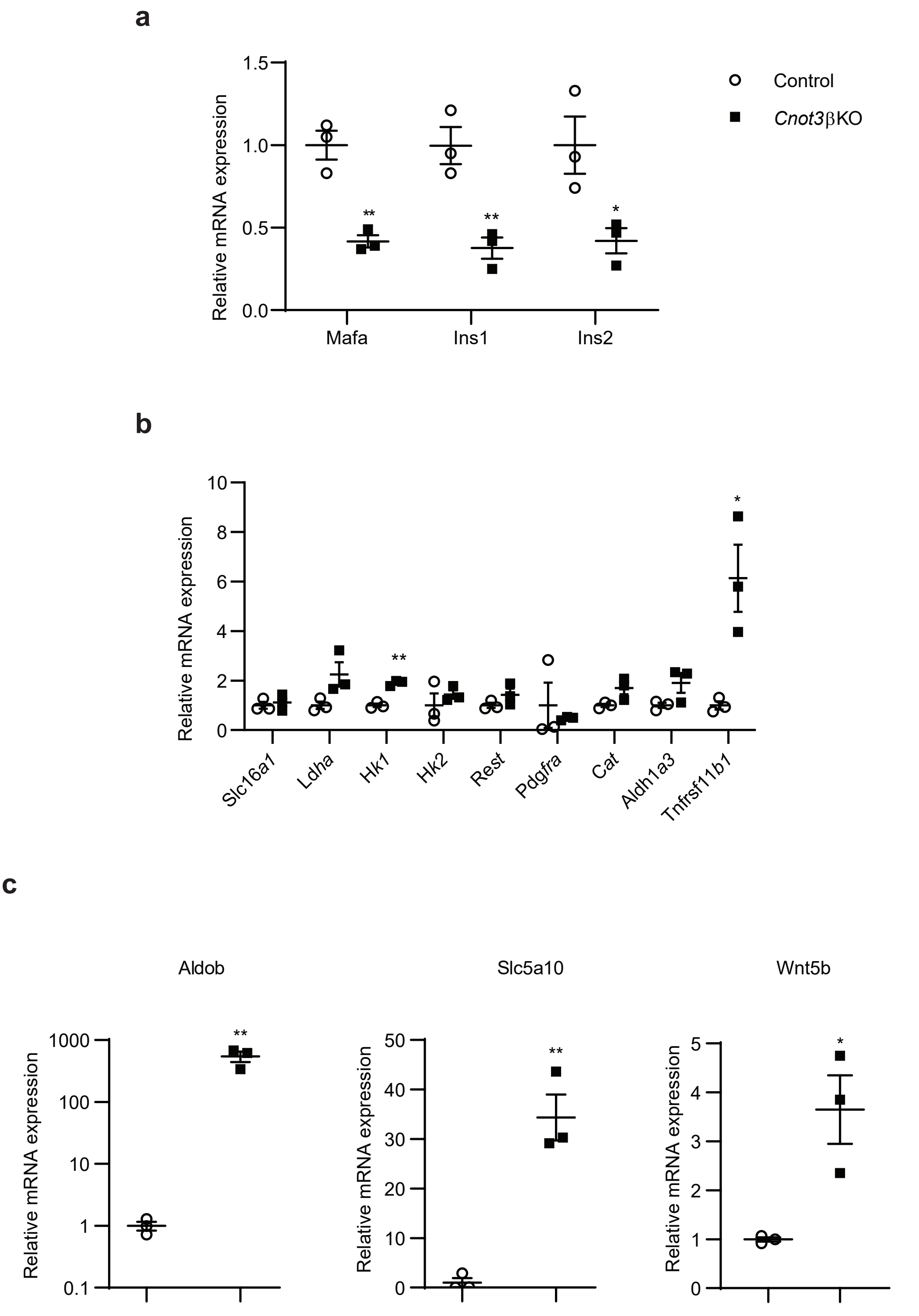

Supplementray Fig. 12:

(a) qPCR analysis of *Mafa*, *Ins1* and *Ins2* in islets isolated from 4-week-old control and *Cnot3*βKO mice (n=3).

(b) qPCR analysis of the indicated genes in islets isolated from 4- week-old control and *Cnot3*βKO mice, only *Hk1* and *Tnfrsf11b1* displayed a significantly increased expression (n=3).

(c) qPCR analysis of top upregulated and stablized genes as inferred from RNA-seq and global stability analyses (n=3).

Supplementary Tables:

Supplementary Table 1: Upregulated and stabilized genes in *Cnot3*βKO that are upregulated in *db/db* islets:

Aldob	Dync1i1	Maged2	Rnh1
Klhl38	Serpinf1	Nudt18	Ippk
Cidea	Fbxo16	Asap1	Sgsm3
Slc5a10	Itpr2	Fxyd3	Slco1a6
Slc35f4	Degs2	Golm1	Lrch1
Ramp1	Smpdl3a	Tmem62	Apbb1
Wnt5b	Crmp1	Krt18	Edem2
Krt80	Arhgap6	Lmf1	Snap47
Gm973	Naip2	Krt7	Tmem135
Spon2	Sybu	Tapbpl	Osbpl5
Il1r2	Igsf10	Itgb5	Xpnpep1
Gna14	Trpc4	Sdf2	Acad11
Cpb2	Plet1	Tyw1	
Dapl1	Dsc2	Tmprss4	
Abhd11os	Trim66	Sytl1	
Smoc1	Atl1	Cwc22	
Bambi	Ebpl	Ildr1	
Akap6	Vil1	Ggt7	
Fbxo32	Cd81	Tbrg1	
Acer2	Efhc2	Dhx40	
Angptl2			

Supplementary Table 2: Primers used for genotyping

Gene	Primers
Cnot3-conditional	
N3 FW	CCAGTCTATCTGATGTGGAATTCCTCCATG
N3 RV	AGGCTGGCAGCTCCTGGAAAGGCTAAGAGG
Cre	
Cre FW	TCGATGCAACGAGTGATGAG
Cre RV	TTCGGCTATACGTAACAGGG
Il-2 (internal control for Cre)	
Il-2 FW	CTAGGCCACAGAATTGAAAGATCT
Il-2 RV	GTAGGTGGAAATTCTAGCATCATCC
mTmG	
mTmG FW	CTCTGCTGCCTCCTGGCTTCT
mTmG RV	CGAGGCGGATCACAAGCAATA
mTmG Mut RV	TCAATGGGCGGGGGTCGTT

Supplementary Table 3: Primary antibodies used for immunoblot analysis

Antibody	dilution	Manufacturer (Cat#)
CNOT1	1:1000	Proteintech (14276-1-AP)
CNOT2	1:1000	Proteintech (34214)
CNOT3	1:1000	Bio Matrix Research Incorporation
CNOT6L	1:1000	Produced in collaboration with Bio Matrix Research Incorporation
CNOT7	1:1000	Abnova (H00029883-M01)
CNOT8	1:1000	Produced in collaboration with Bio Matrix Research Incorporation

CNOT9	1:1000	Proteintech (22503-1-AP)
GAPDH	1:2000	Cell Signaling (2118L)
α-tubulin	1:1000	Sigma (T9026)
ALDH1A3	1:1000	Novus biologicals (NBP2-15339)
MCT1	1:30	Novus biologicals (NBP1-59656)
LDHA	1:30	Cell Signaling (2012)
ALDOB	1:1000	Proteintech (18065-1-1AP)

Supplementary Table 4: Secondary antibodies used for immunoblot analysis

Antibody	dilution	Manufacturer (Cat#)
Anti-Rabbit HRP	1:3000	GE healthcare (NA934V)
Anti- mouse HRP	1:3000	GE healthcare (NA931V)
Anti-mouse HRP (light chain specific)	1:6000	Jackson immunoresearch laboratories (115-035-174)

Supplementary Table 5: Primary antibodies used for immunofluorescence analysis

Antibody	dilution	Manufacturer (Cat#)
CNOT3	1:100	Bio Matrix Research Incorporation
EGFP	1:100	Clontech (632569)
Insulin	No dilution	Agilent (IR00261)
GLUC	1:500	Phoenix pharmaceuticals (H-028-05)
SST	1:100	Sigma-Aldrich (SAB4502861)

PPT	3µg/ml (no antigen retrieval)	Sigma-Aldrich (SAB2500747)
SYP	1:500	Abcam (ab32127)
MAFA	1:500	Bethyl Laboratories (IHC-00352)
GLUT2	5µg/ml	Abcam (ab54460)

Supplementary Table 6: Secondary antibodies used for immunofluorescence analysis

Antibody	dilution	Manufacturer
Goat-anti-mouse-Alexa-488	1:500	Thermofisher (A-21424)
Goat-anti-rabbit-Alexa-488	1:500	Thermofisher (A-11008)
Goat- anti-guinea pig-647	1:500	Thermofisher (A-21450)
Goat-anti-rabbit-Alexa-555	1:500	Thermofisher (A-21429)
Donkey-anti-goat-568	1:500	Thermofisher (A-11057)

Supplementary Table 7: Primers used for qPCR reactions

Gene	Forward (5'-3')	Reverse (5'-3')
Ins1	GAAGTGGAGGACCCACAAGTG	CTGAAGGTCCCCGGGGCT
Ins2	ATGGCCCTGTGGATGCGCTT	CTAGTTGCAGTAGTTCTCCAGCTGG
Ngn3	GTCGGGAGAACTAGGATGGC	AGTCACCCACTTCTGCTTCG
Aldh1a3	GGGCCTCAGATCGACCAAAA	CTAGCTTGGCCCCTTCCTTC
Myt1	TATGCTTGCCCCAAAGATTC	GGCTTTGTGCTGAGGTTCTC
Dcx	GCTGACTCAGGTAACGACCA	ACAGGTCTACCTTGTGCTTCC
Nes	CCCTTAGTCTGGAAGTGGCT	CTTCAGCTTGGGGTCAGGAAAG

Tnfrsf11b	GTTTCCCGAGGACCACAAT	CCATTCAATGATGTCCAGGAG
Mafa	CAAGGAGGAGGTCATCCGAC	TCTCCAGAATGTGCCGCTG
Nkx2.2	CGCTACAAGATGAAACGTGCC	CACCTTGCGGACACTATGGG
Nkx6.1	GAGAGTCAGGTCAAGGTCTGGT	TCCGAGTCCTGCTTCTTCTTG
NeuroD1	TCCCTACTCCTACCAGTCCC	CTGGTGCAGTCAGTTAGGGG
Pdx1	CAGTGGGCAGGAGGTGCTTA	GGGCCGGGAGATGTATTTGTT
Slc2a2	ATTACCGACAGCCCATCCTC	AGCACAGAGACAGCCGTGAA
Pcx	GGGCGGAGCTAACATCTACC	TATACTCCAGACGCCGGACA
Gck	CTGTTAGCAGGATGGCAGCTT	TTTCCTGGAGAGATGCTGTGG
Cpe	GGTACTGCTCACGAATACAGTTCC	CTGCTCAGGTAATTGAAGTCTTGC
Pcsk1	GACCTGCACAATGACTGCAC	GGTCCAGACAACCAGATGCT
Pcsk2	GTTTTTGCGTCTGCCGAGAG	CAAAGGGGAGCTTTCGGACT
Abcc8	TCAACTTGTCTGGTGGTCAGC	GAGCTGAGAAAGGGTCATCCA
Ucn3	GCTGTGCCCCTCGACCT	TGGGCATCAGCATCGCT
Slc30a8	CAGAGAACTTCGACAGAAGCC	CTTGCTTGCTCGACCTGTT
Cacna1c	ATGAAAACACGAGGATGTACGTT	ACTGACGGTAGAGATGGTTGC
Cacna1d	GAAGCTGCTTGACCAAGTTGT	AACTTCCCCACGGTTACCTC
Cacnb2	GCAGGAGAGCCAGATGGA	TCCTGGCTCCTTTTCCATAG
Slc16a1	GCTTGGTGACCATTGTGGAAT	CCCAGTACGTGTATTTGTAGTCTCCAT
Ldha	ATGAAGGACTTGGCGGATGA	ATCTCGCCCTTGAGTTTGTCTT
Hk1	GTGGACGGGACGCTCTAC	TTCACTGTTTGGTGCATGATT
Hk2	GCCTCGGTTTCTCTATTTGGC	ATACTGGTCAACCTTCTGCACT
Rest	GCGCACAGTTCAGAGGAGT	CATGTTGGCACTGTTGTTGA

Pdgfra	GCGAGTTTAATGTTTATGCCTTG	GGCACAGGTCACCACGAT
Aldob	AGAAGGACAGCCAGGGAAAT	GTTCAGAGAGGCCATCAAGC
Slc5a10	ACCTACACTGTGTGTCCCT	CGGCCACATTCAAGGCAAAAT
Wnt5b	AGCACCGTGGACAACACAT	AAGGCAGTCTCTCGGCTACC
Cat	TGCCCCCAACTATTACCCCA	TCCGCACCTGAGTGACATTG
Abtb2	CGCTGAGCACGGTTACCT	CAGCTTCCAGGGAACACC
Fgf1	TTATACGGCTCGCAGACACC	TCTGGCCATAGTGAGTCCGA
Yap1	AAATGCTCCAAAATGTCAGGA	CATTCGGAGTCCCTCCATC
Igfbp4	AAGATCGTGGGGACACCTC	GTGGGTACGGCTCTGTGAG
Acot7	AGGTGCCTCCCATTGTGTAT	TTCTGGGCTTCATAGCGTTT
Cxcl12	CCCTGCCGGTTCTTCGA	CAGCCGTGCAACAATCTGAA
Gapdh	CTGCACCACCAACTGCTTAG	GTCTTCTGGGTGGCAGTGAT

Supplementary Scripts:

Script for differential expression analysis:

```
# Load data for DE
raw_counts=read.csv("all raw counts.csv", stringsAsFactors=FALSE)


#Download edger for R version 3.6.1
if (!requireNamespace("BiocManager", quietly = TRUE))
  +    install.packages("BiocManager")
BiocManager::install("edgeR")


#Load edgeR
library("edgeR")


#set working directory




#Read in data
counts <- raw_counts[ , -1]
rownames(counts)<-raw_counts[ , 1] #gene Ensembl IDs
View(counts)


#convert count matrix to edgeR DGElist Object


group<- c(rep("Control",3), rep("KO",3))
cds<-DGEList(counts, group=group)
names(cds)
#original count data
head(cds$counts)
#contains sample information
```

```
head(cds$samples)

# How many genes have 0 counts across all samples

sum( cds$all.zeros )



##Filter out low count reads keep only genes with at least 1 read per million reads in at least 3 samples

#once this is done, can calculate normalisation factors which correct for different compositions of samples

#effective library size = product of actual library size and these factors


countsPerMillion <- cpm(cds, normalized.lib.sizes=TRUE)

summary(countsPerMillion)

#'summary' is a useful function for exploring numeric data; eg. summary(1:100)

countCheck <- countsPerMillion > 1

head(countCheck)

keep <- which(rowSums(countCheck) >= 3)

cds <- cds[keep,]

summary(cpm(cds))


cds <- calcNormFactors( cds )

cds$samples


# effective library sizes

cds$samples$lib.size * cds$samples$norm.factors



##an MD plot can show the performance of the TMM normalization, visualizes the library size-adjusted log-fold change between two

#libraries (the difference) against the average log-expression across those libraries (the mean)
```

```
#The following MD plot is generated by comparing sample 1 against an artificial library
#constructed from the average of all other samples.
options(max.print=100000)
plotMD(cpm(cds, log=TRUE), column=1)
abline(h=0, col="red", lty=2, lwd=2)
#Ideally, the bulk of genes should be centred at a log-fold change of zero. This indicates
#that any composition bias between libraries has been successfully removed. This quality
#check should be repeated by constructing a MD plot for each sample. column =1 means smaple 1.


#Multi-Dimensional Scaling (MDS) Plot
#For a few samples, the MDS plot is an appropriate 2D non-linear multidimensional analysis extrapolation.
plotMDS( cds , main = "MDS Plot", labels = colnames( cds$counts ) )


#Estimating Dispersions
#1st: calculate common dispersion
#each gene gets assigned the same dispersion estimate
#output of the estimation includes estimate and other elements added to object cds
cds <- estimateCommonDisp( cds )
names( cds )
#The estimate
cds$common.dispersion

#2nd calculate Tagwise dispersion
#with common dispersion, can estimate tagwise dispersions
#each gene will get its own dispersion estimate
#Tagwise dispersion
```

```
cds <- estimateTagwiseDisp( cds)

names( cds )

summary( cds$tagwise.dispersion)



#Testing

# exactTest() performs pairwise tests for diff exp between 2 groups

# pair indicates which groups should be compared

# output is a list of elements, one of which is a table of results

de.tgw <- exactTest( cds , dispersion="tagwise", pair = c( "Control" , "KO" ) )



# Back to count matrix for tagwise analysis

# Store full topTags results table

resultsTbl.tgw <- topTags( de.tgw , n = nrow( de.tgw$table ) )$table

head( resultsTbl.tgw )

# Names/IDs of DE genes

de.genes.tgw <- rownames( resultsTbl.tgw )[ resultsTbl.tgw$PValue <= 0.05 ]

# Up/Down regulated summary for tagwise results

summary( decideTestsDGE( de.tgw , p.value = 0.05 ) ) # the adjusted p-values are used here



#Output results

#make a table or csv file containing results with concentrations, fold-changes, p-values
```

```
#up/down regulated variable, dispersions, and the count matrix

# Change column names to be specific to the analysis, logConc and logFC are the same in both.

colnames( resultsTbl.tgw ) <- c( "logFC" , "logConc" , "pVal.Tgw" , "adj.pVal.Tgw" )
# Below provides the info to re-order the count matrix to be in line with the order of the results.
wh.rows.tgw <- match( rownames( resultsTbl.tgw ) , rownames( cds$counts ) )


# Tagwise Results
combResults.tgw <- cbind( resultsTbl.tgw ,
            "Tgw.Disp" = cds$tagwise.dispersion[ wh.rows.tgw ] ,
            "UpDown.Tgw" = decideTestsDGE( de.tgw , p.value = 0.05 )[ wh.rows.tgw ] ,
            cds$counts[ wh.rows.tgw , ] )
head( combResults.tgw )

# Ouput csv tables of results
write.table( combResults.tgw , file = "combResults_tgw_ex1.csv" , sep = "," , row.names = TRUE )

# Add gene names to results csv
combResults_tgw_ex1 <- read.csv("combResults_tgw_ex1.csv", stringsAsFactors=FALSE)
combResults_tgw_ex1$Gene_ID <- row.names(combResults_tgw_ex1)
gene_ref <- read.csv("gene_ref.csv", stringsAsFactors=FALSE)

final<- merge(combResults_tgw_ex1, gene_ref, by = "Gene_ID")
write.csv(final, file = "Final.csv", row.names = FALSE, quote = FALSE )

# find number of genes above and below cutoff
sum(final$logFC >= 1)
```

```
sum(final$logFC <= -1)


#get list of significanlty up regulated
final_up <- final[final$UpDown.Tgw == 1,]
write.csv(final_up, file = "final_up.csv", row.names = FALSE, quote = FALSE )



#get list of significanlty down regulated
final_down <- final[final$UpDown.Tgw == -1,]
write.csv(final_down, file = "final_down.csv", row.names = FALSE, quote = FALSE )



#draw volcano plot:
final=read.table(file.choose(), sep = ",", header = TRUE)
with(final, plot(logFC, -log10(pVal.Tgw), pch=20, main="Volcano plot", xlim=c(-2.5,2), ylim=c(0,30)))
# Add colored points: red if padj<0.05, FC>2, blue ifpadj<0.05, FC>2
with(subset(final, adj.pVal.Tgw<.05 & logFC>1), points(logFC, -log10(pVal.Tgw), pch=20, col="red"))
with(subset(final, adj.pVal.Tgw<.05 & logFC<(-1)), points(logFC, -log10(pVal.Tgw), pch=20, col="blue"))
```

Script for transcriptomic and proteomic correlation analysis:

```
#prepare two files with DE genes (TR) and DE proteins (MS) with ensemble gene ID and logFC only
#Upload files
MS<- read.csv("MS.csv", stringsAsFactors = FALSE)
TR<- read.csv("TR.csv", stringsAsFactors = FALSE)

MS2 <- subset(MS, MS$Gene_ID %in% TR$Gene_ID)
TR2 <- subset(TR, TR$Gene_ID %in% MS$Gene_ID)

all_MS_TR_common<-merge(MS2,TR2, by="Gene_ID")
write.csv(all_MS_TR_common, "All_MS_TR_common.csv", row.names = FALSE, quote= FALSE)

#Correlation analysis
install.packages("ggpubr")
library(ggpubr)

ggscatter(all_MS_TR_common, x = "MS_logFC", y = "TR_logFC",
        add = "reg.line", conf.int = TRUE,
        cor.coef = TRUE, cor.method = "pearson",
        xlab = "Proteomic log2FC", ylab = "Transcriptomic log2FC")
```

Script for mRNA stability analysis:

Previously published in [40]:

```
# load edgeR library

library(edgeR)


# input files and parameters

exsFile <- "exon_reads.txt"  #exons raw counts file


conditions <- c("Control","Control","Control","KO", "KO", "KO") # correspond to the columns in exsFile and insFile; the first condition will be the reference


# read in count tables and remove the first column (ensembl ID)

cntEx <- read.delim(exsFile)[,-1]


# combine exon and intron raw counts for edgeR containing genes with sufficient counts in both exonic and intronic levels

raw_counts <- read.csv("Cnt.csv", stringsAsFactors=FALSE)  # Cnt is combined exons and introns file 240319 keeping only normalized exons and introns and ensembl IDs

Cnt <- raw_counts[ , -1]

rownames(Cnt)<-raw_counts[ , 1] #gene Ensembl IDs

View(Cnt)


# edgeR workflow

factorRegion    <- factor(rep(c("ex","in"),each=ncol(cntEx)), levels=c("in", "ex")) # define experimental factor exon/intron

factorCondition <- factor(rep(conditions,2), levels=unique(conditions)) # define experimental factor 'conditions'


y <- DGEList(counts=Cnt, genes=data.frame(EnsemblID=rownames(Cnt))) # define DGEList object

y <- calcNormFactors(y) # determine normalization factors
```

```
design <- model.matrix(~ factorRegion * factorCondition) # design matrix with interaction term
rownames(design) <- colnames(Cnt)

y <- estimateDisp(y, design) # estimate dispersion
fit <- glmFit(y, design) # fit generalized linear model
lrt <- glmLRT(fit) # calculate likelihood-ratio between full and reduced models
#final table with significance level for each gene
tt <- topTags(lrt, n=nrow(y))
head(tt$table)
write.csv(tt$table, file = "tt.csv", row.names = FALSE, quote = FALSE )

stablitytable <- read.csv("tt.csv", stringsAsFactors=FALSE)
#to add gene symbols to table
gene_ref <- read.csv("gene_ref.csv", stringsAsFactors=FALSE)

final<- merge(stablitytable, gene_ref, by = "Gene_ID")
write.csv(final, file = "Final stability.csv", row.names = FALSE, quote = FALSE )
```
