# Supplementary material for: Loss of β-cell identity and diabetic phenotype in mice caused by disruption of CNOT3-dependent mRNA deadenylation
Source: Commun Biol. 2020 Aug 28;3:476. doi: 10.1038/s42003-020-01201-y (PMC7455721; doi:10.1038/s42003-020-01201-y)
Supplement: Supplementary file 2 — Description of Additional Supplementary File [file 42003_2020_1201_MOESM2_ESM.pdf]

Description of additional supplementary file

Supplementary Data 1: Source data for the main and supplementary figures.
