# Supplementary material for: Loss of β-cell identity and diabetic phenotype in mice caused by disruption of CNOT3-dependent mRNA deadenylation
Source: Commun Biol. 2020 Aug 28;3:476. doi: 10.1038/s42003-020-01201-y (PMC7455721; doi:10.1038/s42003-020-01201-y)
Supplement: Supplementary file 4 — Reporting Summary [file 42003_2020_1201_MOESM4_ESM.pdf]

nature research

Corresponding author(s): Tadashi Yamamoto

Last updated by author(s): Jul 7, 2020

Reporting Summary

Nature Research wishes to improve the reproducibility of the work that we publish. This form provides structure for consistency and transparency in reporting. For further information on Nature Research policies, see Authors & Referees and the Editorial Policy Checklist .

Statistics

For all statistical analyses, confirm that the following items are present in the figure legend, table legend, main text, or Methods section.

n/a	Confirmed	
[ ]	[x]	The exact sample size (*n*) for each experimental group/condition, given as a discrete number and unit of measurement
[ ]	[x]	A statement on whether measurements were taken from distinct samples or whether the same sample was measured repeatedly
[ ]	[x]	The statistical test(s) used AND whether they are one- or two-sided<br>*Only common tests should be described solely by name; describe more complex techniques in the Methods section.*
[ ]	[x]	A description of all covariates tested
[x]	[ ]	A description of any assumptions or corrections, such as tests of normality and adjustment for multiple comparisons
[ ]	[x]	A full description of the statistical parameters including central tendency (e.g. means) or other basic estimates (e.g. regression coefficient) AND variation (e.g. standard deviation) or associated estimates of uncertainty (e.g. confidence intervals)
[x]	[ ]	For null hypothesis testing, the test statistic (e.g. *F*, *t*, *r*) with confidence intervals, effect sizes, degrees of freedom and *P* value noted<br>*Give P values as exact values whenever suitable.*
[x]	[ ]	For Bayesian analysis, information on the choice of priors and Markov chain Monte Carlo settings
[x]	[ ]	For hierarchical and complex designs, identification of the appropriate level for tests and full reporting of outcomes
[x]	[ ]	Estimates of effect sizes (e.g. Cohen's *d*, Pearson's *r*), indicating how they were calculated

Our web collection on statistics for biologists contains articles on many of the points above.

Software and code

Policy information about availability of computer code

Data collection	doesn't apply
Data analysis	RNA sequencing data analysis: Paired-end RNA-seq data were mapped to the Mus musculus reference strain mm10 UCSC using Strand NGS, next generation sequencing analysis software. Counts for each sample were imported into the RStudio (v.1.1.453). Genes without an expression level of at least one read per million mapped reads in at least three samples were removed before differential gene expression testing between control, and Cnot3βKO islet RNA replicates, using the edgeR function in the Bioconductor package edgeR. GO analysis using DAVID annotation tool (https://david.ncifcrf.gov/). Softwares for statistical analysis: GraphPad Prism (GraphPad Software v.8.0), and GSEA (v.4.0.1) developed by Broad Institute (http://software.broadinstitute.org/gsea/index.jsp). Softwares for images analysis and processing: Fiji (www.fiji.sc) and Imaris (v.2.9) softwares, respectively.

For manuscripts utilizing custom algorithms or software that are central to the research but not yet described in published literature, software must be made available to editors/reviewers. We strongly encourage code deposition in a community repository (e.g. GitHub). See the Nature Research guidelines for submitting code & software for further information.

Data

Policy information about availability of data

All manuscripts must include a data availability statement. This statement should provide the following information, where applicable:

- Accession codes, unique identifiers, or web links for publicly available datasets
- A list of figures that have associated raw data
- A description of any restrictions on data availability

The raw transcriptomics data sets described in the current study are available through ArrayExpress under the accession number [E-MTAB-8729]. The mass spectrometric proteomics data have been deposited to the ProteomeXchange Consortium via the PRIDE partner repository with the dataset identifier PXD018403. The authors declare that all data supporting the findings of this study are available within the paper and its supplementary information files or can be obtained from the authors upon reasonable request.

Field-specific reporting

Please select the one below that is the best fit for your research. If you are not sure, read the appropriate sections before making your selection.

[x] Life sciences [ ] Behavioural & social sciences [ ] Ecological, evolutionary & environmental sciences

For a reference copy of the document with all sections, see nature.com/documents/nr-reporting-summary-flat.pdf

Life sciences study design

All studies must disclose on these points even when the disclosure is negative.

Sample size	N/A
Data exclusions	We didn't exclude any data.
Replication	We used at least 3 biological replicates for each experiment. Only bulk poly (A) tail experiment was done once on RNA extracted from WT and KO islets each pooled from 4 mice per group. We didn't repeat the experiment as the results were consistent with previous reports and each sample was pooled from 4 biological replicates.
Randomization	The experiments were not randomized.
Blinding	The investigators were not blinded to allocation during experiments and outcome assessment.

Reporting for specific materials, systems and methods

We require information from authors about some types of materials, experimental systems and methods used in many studies. Here, indicate whether each material, system or method listed is relevant to your study. If you are not sure if a list item applies to your research, read the appropriate section before selecting a response.

Materials & experimental systems

n/a	Involved in the study	
[ ]	[x]	Antibodies
[ ]	[x]	Eukaryotic cell lines
[x]	[ ]	Palaeontology
[ ]	[x]	Animals and other organisms
[x]	[ ]	Human research participants
[x]	[ ]	Clinical data

Methods

n/a	Involved in the study	
[x]	[ ]	ChIP-seq
[x]	[ ]	Flow cytometry
[x]	[ ]	MRI-based neuroimaging

Antibodies

Antibodies used	CNOT1 (Proteintech (14276-1-AP)); CNOT2 (Proteintech (34214)); CNOT3 (clone 54, Produced in collaboration with Bio Matrix Research Incorporation); CNOT6L (Clone 615, Produced in collaboration with Bio Matrix Research Incorporation); CNOT7 (Abnova (H00029883-M01)); CNOT8 (Produced in collaboration with Bio Matrix Research Incorporation); CNOT9 (Proteintech (22503-1-AP)); GAPDH (Cell Signaling (2118L)); α-tubulin (Sigma (T9026)); ALDH1A3 (Novus biologicals (NBP2-15339)); MCT1 (Novus biologicals (NBP1-59656)); LDHA (Cell Signaling (2012)); ALDOB (Proteintech (18065-1-1AP)); Anti-Rabbit HRP (GE healthcare (NA934V)); Anti- mouse HRP (GE healthcare (NA931V)); EGFP (Clontech (632569)); Insulin (Agilent (IR00261)); Glucagon (Phoenix pharmaceuticals (H-028-05)); Somatostatin (Sigma-Aldrich (SAB4502861)); Pancreatic Polypeptide (Sigma-Aldrich (SAB2500747)); Synaptophysin (Abcam (ab32127)); MafA (Bethyl Laboratories (IHC-00352)); GLUT2 (Abcam (ab54460)); Goat-anti-mouse-Alexa-488 (Thermofisher (A-21424)); Goat-anti-rabbit-Alexa-488 (Thermofisher (A-11008)); Goat- anti-guinea pig-647 (Thermofisher (A-21450)); Goat-anti-rabbit-Alexa-555 (Thermofisher (A-21429)); Donkey-anti-goat-568 (Thermofisher (A-11057)).
Validation	All CCR4-NOT complex subunits antibodies were validated in KO mice or cells in previous publications.

Eukaryotic cell lines

Policy information about cell lines

Cell line source(s)	MIN6-m9 cells were obtained from Dr. Susumu Seino, Kobe University Graduate School of medicine.

Authentication	Minami K, Yano H, Miki T, Nagashima K, Wang C-Z, Tanaka H, et al. Insulin secretion and differential gene expression in glucose-responsive and -unresponsive MIN6 sublines. American Journal of Physiology-Endocrinology and Metabolism. 2000;279(4):E773-E81.
Mycoplasma contamination	Mycoplasma contamination was not detected with DAPI staining. We further confirmed the absence of Mycoplasma contamination using e-Myco plus Mycoplasma PCR detection kit.
Commonly misidentified lines (See ICLAC register)	N/A

Animals and other organisms

Policy information about studies involving animals; ARRIVE guidelines recommended for reporting animal research

Laboratory animals	Adult male mice. 4-12 weeks of age. Cnot3 flox/flox mice , Ins1 -cre mice (Riken Bioresource Center, # RBRC09525) , mTmG mice (Jackson Laboratory, # 007676) with C57BL/6J background. +/db and db/db mice (Jackson Laboratory, #000697).
Wild animals	The study didn't involve wild animals.
Field-collected samples	The study didn't contain field collected samples.
Ethics oversight	All mouse experiments were approved by the Animal Care and Use Committee of Okinawa Institute of Science and Technology (OIST) Graduate University, Okinawa, Japan.

Note that full information on the approval of the study protocol must also be provided in the manuscript.
